# Occupational stress among nurses and pre-hospital emergency staff: application of fuzzy analytic hierarchy process (FAHP) method

**DOI:** 10.17179/excli2018-1505

**Published:** 2018-08-20

**Authors:** Fazel Rajabi, Mehdi Jahangiri, Hossein Molaeifar, Marzieh Honarbakhsh, Payam Farhadi

**Affiliations:** 1 Student Research Committee, School of Health, Shiraz University of Medical Sciences, Shiraz, Iran; 2 Research Center for Health Science, Institute of Health, Associate Professor, Department of Occupational Health, Shiraz University of Medical Sciences; 3 Department of Occupational Health, Larestan University of Medical Sciences, Larestan, Iran; 4 Department of Management, Zand Higher Education Institute, Shiraz, Iran

**Keywords:** occupational stress factors, nurses, pre-hospital emergency staff, FAHP method

## Abstract

Healthcare professionals, especially nurses and pre-hospital emergency (PHE) staff, are influenced by many stressors due to their responsibility to provide comfort as well as care and treatment of patients. The aim of the present study was to identify and rank the occupational stressors in nurses and PHE staff using Fuzzy Analytic Hierarchy Process (FAHP) method. In this cross-sectional study, occupational stress factors in nurses and PHE staffs were identified and ranked by 30 experts, using FAHP method. Occupational stress factors were collected by General Health Questionnaire (GHQ), Job Stress Questionnaires as well as a literature review. Among the occupational stress factors in nurses, the highest scores were related to “Incompatibility between work schedule and life conditions” (0.03986) and “Being criticized by supervisors” (0.03723), respectively. The most common stress factors in PHE staff were related to “Care of patients with critical health conditions” (0.07258), “High number of missions” (0.07056), respectively. The overall results of this study showed that managerial factors and factors related to patient care are the most important causes of occupational stress among nurses and PHE staff. These factors should be considered in the implementation of control strategies for reducing and managing occupational stress.

## Introduction

Today, stress is taken into consideration by doctors, psychologists, behavioral and management scientists as the most important factor causing mental, physical and behavioral disorders (Pashib et al., 2015[42]). The National Institute for Occupational Health and Safety (NIOSH) has defined occupational stress as a harmful physical and emotional response that occurs when job requirements are not consistent with employees' abilities and needs (DHHS, 2008[14]). Previous studies have shown about 30 percent of workforce in developed countries suffer from occupational stress and this number is higher in newly industrialized and developing countries. Just in America, about 11 million people suffer from occupational stress (Hoel et al., 2001[20]; Nazari et al., 2015[38]). Occupational stress has physical, mental and behavioral complications. Physical complications include cardiovascular, gastrointestinal, musculoskeletal and immune system disorders, various cancers and increased incidence of injuries and accidents. Behavioral outcomes of occupational stress include work absenteeism, smoking, sleep disorders, alcohol and drug abuse, and addiction (LaDou and Harrison, 2007[28]; Yaribeygi et al., 2017[57]). In addition, chronic exposure to occupational stressors can cause occupational burnout syndrome. Burnout has three sub-dimensions of emotional exhaustion, depersonalization and personal accomplishment (Tuna and Baykal, 2014[53]; Nazari et al., 2016[39]).

The evidence showed that the type of work can have a major role in employees' stress (Adriaenssens et al., 2017[1]). Medical-related professions such as nursing and pre-hospital emergency are influenced by various stressors due to their responsibility to provide comfort and convenience for patients as well as their care and treatment (Motie et al., 2010[37]; Rahmani et al., 2010[44]; Jahangiri et al., 2016[23]). Nurses constitute that 80 percent of employees in Iran health care system and 80 percent of workload in this system is laid upon them (Hazavehei et al., 2017[19]). They experience a wide range of occupational stress because of their work type, skill, emotional burden and full care of patients (McVicar, 2003[36]). Occupational stress has been identified as one of the five causes of turnover among nurses (Letvak and Buck, 2008[31]).

In order to protect health of individuals, disaster management and medical emergency staff (Pre-hospital) are responsible for providing health services to patients in an emergency and, if necessary, transporting them to medical centers. This profession is also one of the most stressful jobs for some reasons including time pressure, patients' critical situation, patient's companions expectations, open workplace, fear of incompetency in saving dying patients, decision-making in critical situations (Scullion, 1992[47]). Previous studies showed that occupational burnout, an advanced and chronic form of occupational stress, is very common in nurses and PHE staff (Jalili et al., 2013[24]; Tuna and Baykal, 2014[53]; Howlett et al., 2015[22]; Nazari et al., 2016[39]). Since the causes of occupational stress and burnout are similar, identifying stressors can be used to identify pro-active strategies for coping with occupational stress and burnout (Chou et al., 2014[11]; Bagnall et al., 2016[6]; Nazari et al., 2016[39]; Mattei et al., 2017[32]).

Considering the critical role of nurses and pre-hospital emergency technicians in healthcare system, it is necessary to determine the exposure to these factors and work pressures and to reduce exposure to stressors. Given the significance of the issue, implementation of control measures may, to some extent, resolve stress-induced problems. On the other hand, since financing the implementation of all stress reduction techniques is not possible, it is required to rank the factors. This study was conducted with the aim of prioritizing the ways of reducing exposure to occupational stressor in nurses and pre-hospital emergency (PHE) staff. In this study, uncertainty of the judgment is involved in the decision-making process using a fuzzy number.

## Materials and Methods

In this qualitative cross-sectional study, at first occupational stressors in nursing and PHE staff were extracted from previous studies to be ranked and evaluated by experts. Nurses' stressors were adapted from related articles (Gray-Toft and Anderson, 1981[16]; Tyson et al., 2002[54]; Lambert et al., 2004[29]; Rothmann et al., 2006[45]; Sveinsdóttir et al., 2006[49]; Noorian et al., 2010[41]; Rahmani et al., 2010[44]; Torshizi and Ahmadi, 2011[52]; Wang and Kong, 2011[56]; Dagget et al., 2016[13]; Johan et al., 2017[25]) and organized as five main dimensions and 51 sub-dimensions. For PHE staff, in addition to related articles (Hawley, 1992[18]; Essex and Scott, 2008[15]; Nirel et al., 2008[40]; Motie et al., 2010[37]; Tehrani et al., 2012[51]; Akbar Aghaeinejad et al., 2014[2]), the revised version of nurses' job stressors questionnaire was also considered and occupational stressors extracted as five main dimensions and 30 sub-dimensions.

In the next stage, identified stressors in the first phase, were prepared in pairwise comparison questionnaires to be evaluated and ranked by 30 experts (15 in nursing and 15 in PHE) using Fuzzy analytic hierarchy process (FAHP). Experts in each job category included five faculty members and 10 supervisors with at least 5 years work experience in nursing or PHE. Each expert compared the stress dimensions and sub-dimensions using the values in Table 1(Tab. 1). 

Consistency of pairwise comparisons was checked by Consistency Ratio (C.R) index through dividing the consistency index by the random consistency ratio matrix (Table 2(Tab. 2), Reference in Table 2: Brunelli, 2014[7]), and the values less than 0.1 were considered as accepted judgments (Hajkowicz et al., 2000[17]; Mazurek, 2017[34]). In cases where the value of the consistency ratio was more than 0.1, the pairwise comparisons were revised. Finally the weight of each dimension and sub-dimension was calculated using Chang's „extent analysis method” (Chang, 1992[8], 1996[9]).

### FAHP method

FAHP is one of the Multi-Criteria Decision Making (MCDM) techniques in the Operations Research (OR) approach which could be applied as a decision support tool when making decisions with various qualitative and quantitative criteria (Çimren et al., 2007[12]; Saaty, 2008[46]). Fuzzy multi-criteria decision-making method is a combination of fuzzy logic and fuzzy multiple attribute decision-making processes (Chang, 1996[9]). Fuzzy sets theory was first proposed by Professor Zadeh in 1965[58], which is used in solving problems in which parameters and values cannot be accurately stated. This approach is a very appropriate tool to deal and get along with uncertainty and modeling of linguistic variables. Its aim is to develop an approximate reasoning by using the fuzzy sets theory in which uncertainty of the judgment is involved in the decision-making process using a fuzzy number (Kwong and Bai, 2002[27]; Honarbakhsh et al., 2018[21]).

The typical MCDM problem deals with the evaluation of a set of alternatives in terms of a set of decision criteria. In these techniques, research is conducted based on the experts' opinion and are more useful in analyzing issues as they are less susceptible to sample size and n<p problem (Number of Variables Exceeds the Number of Observations), and sampling is not necessary to use these techniques. Moreover, the method for data collection in these techniques is a snowball sampling method, in which the theoretical adequacy of the data is important rather than the sample size.

As MCDM techniques are based on expert judgment, and number of qualified experts is usually limited to 5 and 25 (Armacost et al., 1994[4]; Peterson et al., 1994[43]; Mawapanga and Debertin, 1996[33]; Al-Harbi, 2001[3]; Landeta, 2006[30]; Arof, 2015[5]; Kil et al., 2016[26]). In other words, the advantage of these methods is that they do not require a large sample size, and 10 to 15 qualified experts are adequate to prioritize and solve complex problems.

### Chang's extent analysis

Initial version of FAHP which was proposed by two Dutch researchers, van Laarhoven and Pedrycz, in 1983, was based on a logarithmic least squares method (Van Laarhoven and Pedrycz, 1983[55]; Chang, 1996[9]). The complexity of this method led to proposal of another method by Chang in 1996 called „extent analysis method”, where fuzzy logic and fuzzy triangular numbers are applied for AHP pairwise comparisons. The triangular fuzzy number is a fuzzy number that equation of this is as follows (Zadeh, 1965[58]; Chang, 1996[9]):


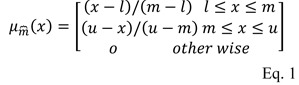


where *l* and *u* stand for the lower and upper limits, respectively; and m is the most probable value of a fuzzy number. Therefore, triangular fuzzy number display by (*l, m, u*). 

If X = {x1, x2,…,xn} be set of object and G = {g1, g2,…,gn} be set of goal, according to extent analysis method, the value of extended analysis m for each object can be calculated as follows:


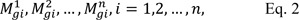


where


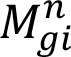


are triangular fuzzy number. The results of this study were calculated from FAHP method by Chang's extended analysis as following steps (Chang, 1996[9]; Honarbakhsh et al., 2018[21]):

**Step 1:** The values of fuzzy synthetic extent (s) for i-th object calculated by Eq. 3: 


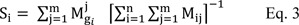


where ⊗ means extensive multiply of two fuzzy numbers and each of obtained fuzzy numbers represents a relative weight of a dimension ratio to another dimension.

To obtain


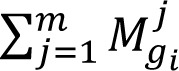


needs to perform fuzzy additional operation as follows:


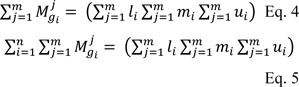


Then the inverse of equation 5 was calculated as follows:


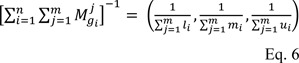


**Step 2:** The degree of possibility computed for


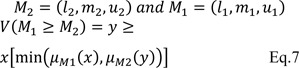


**Figure 1:**
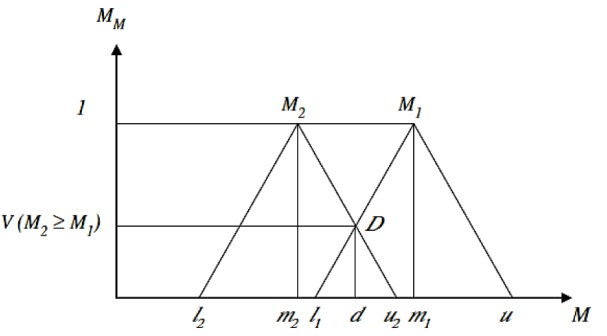
The intersection between M_1_ and M_2 _(Tang and Lin, 2010)

Eq. 7 can be displayed as follows:


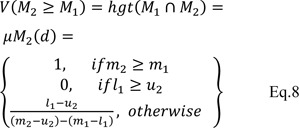


where d is the ordinate of largest intersection point of D between the μM1 and μM2 (Figure 1(Fig. 1), Reference in Figure 1: Tang and Lin, 2010[50]).

**Step 3:** The degree of possibility for convex fuzzy number that are higher than K convex fuzzy number Mi Computed as follows:


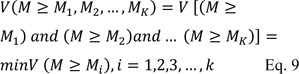


If we suppose equation 10 is true:


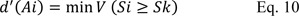


then, the weight vector is obtained as follows:


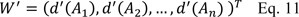


**Step 4:** Finally, the normalized weight of vector was calculated as follows: 


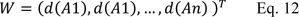


where *W* is non-fuzzy number.

Eventually, total ranking of sub-dimension regardless of their main dimensions was calculated by multiplying the normal weight of main dimensions and normal weight of each sub-dimensions related to the main dimensions.

## Results

**Figure 2:**
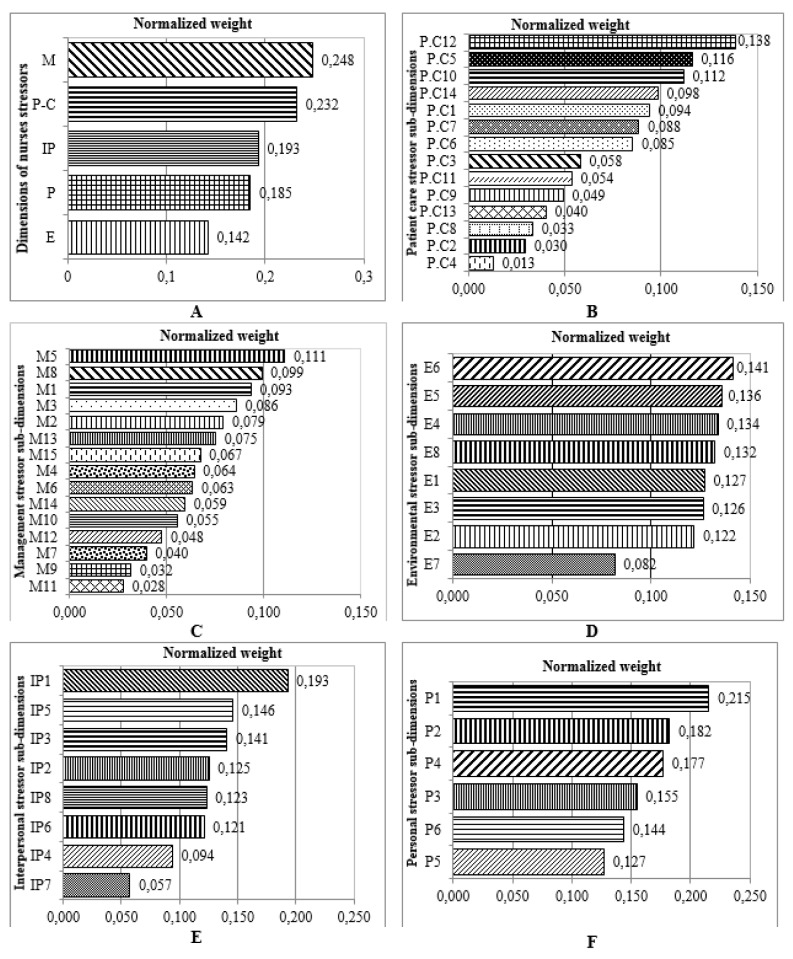
Ranking of occupational stressors from expert nurse’s point of view (FAHP Model) in different dimensions and sub-dimensions (A: dimension; B: patient care stressor; C: management stressor; D: environmental stressor; E: inter personal stressor, F: personal stressor) (refer to Table 3 for information on codes).

Tables 3(Tab. 3) and 4(Tab. 4) show the identified occupational stress factors among nurses and PHE staff, respectively. Figure 2(Fig. 2) shows the results of FAHP weights of occupational stress factors in different dimensions among nurses. As can be seen, the highest and lowest weights were related to managerial and environmental stressor factors, respectively. Regarding patient care, personal, environmental, managerial and interpersonal stressors, maximum weights were related to cardiopulmonary arrest of patients, incompatibility between work schedule and life conditions, lack of opportunity for rest, insufficient pay and being criticized by supervisors in the presence of others, respectively (Figure 2(Fig. 2)).

**Figure 3:**
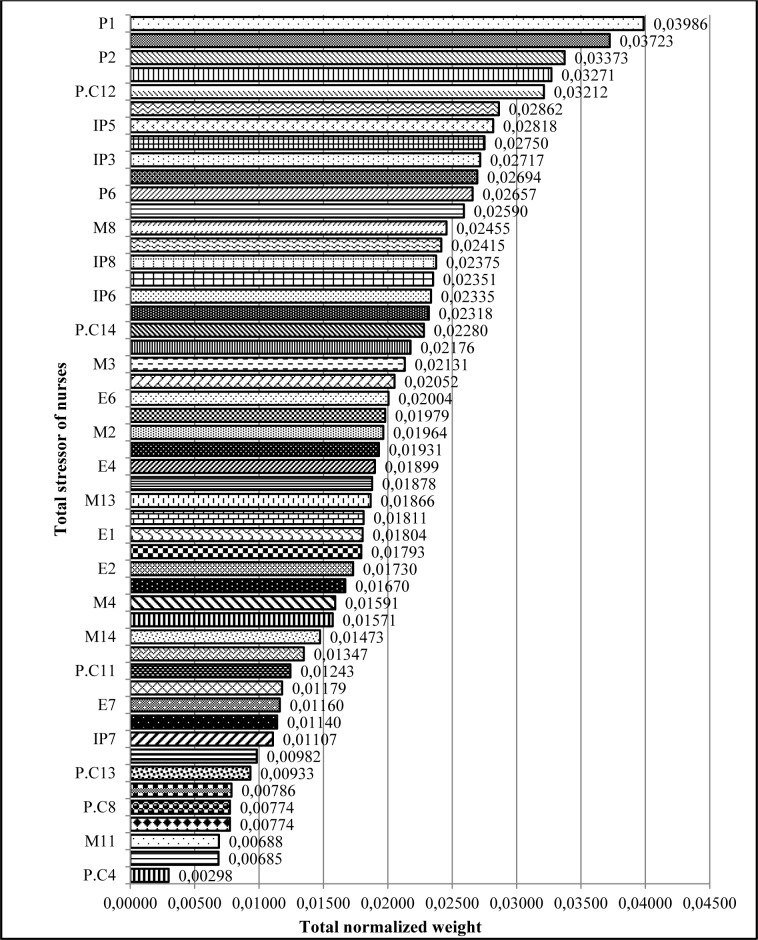
Ranking occupational stressors in nurses regardless of the main categories (dimensions) of stressors using FAHP model (refer to Table 3 for information on codes.)

The overall ranking of stressors in nurses showed that the most common stressors in nurses include „incompatibility between work schedule and life conditions, being criticized by superiors in the presence of others, lack of interest to work in the current ward, inability to making decisions in emergency situations and cardiopulmonary arrest of patients” (Figure 3(Fig. 3)).

**Figure 4:**
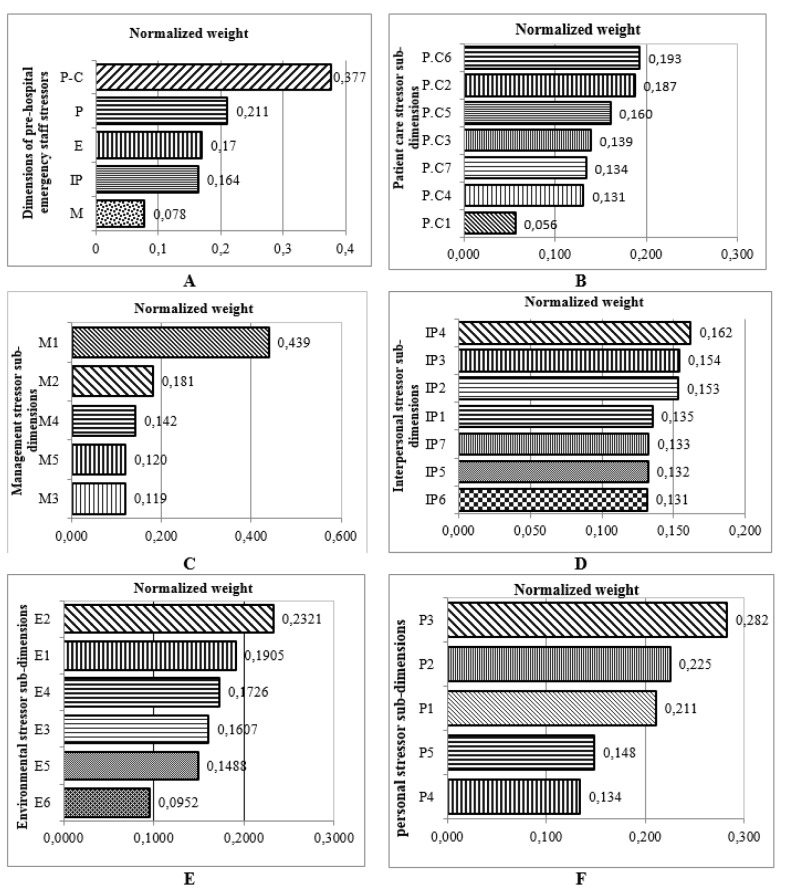
Ranking Occupational stressors dimensions and different sub-dimensions in PHE staff using FAHP model (A: stress dimensions; B: Patient care stressor; C: Management stressor, D: Interpersonal stressor; E: Environmental stressor; F: Personal stressor) (refer to Table 4 for information on codes).

**Figure 5:**
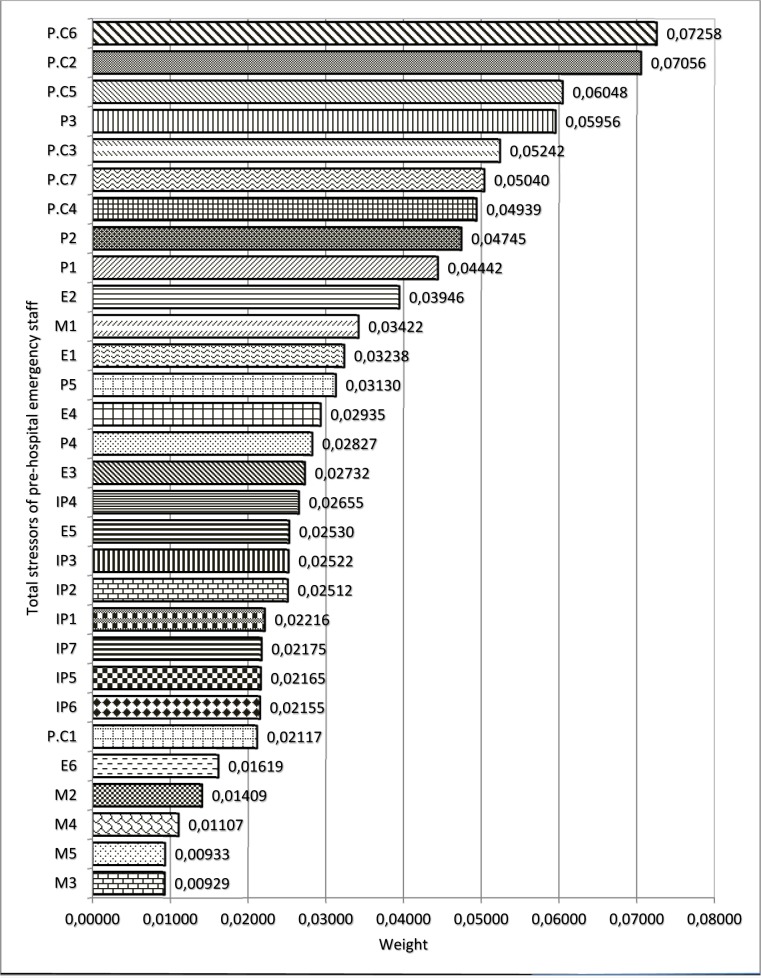
Weight and prioritizing occupational stressors in PHE staff regardless of the main categories (dimensions) of stressors using FAHP model (refer to Table 4 for information on codes).

Figure 4(Fig. 4) (refer to Table 4(Tab. 4) for information on codes) shows the results of FAHP weights of occupational stress factors in different dimensions among PHE staff. As shown, the highest and lowest weight, were related to the patient care and managerial stressors, respectively. Maximum weight in the areas of patient care, personal, interpersonal, environmental and managerial stressors were related to care of patients with critical health conditions, fear of making a mistake in duty, intervention of patient's companions in emergency care, lack of opportunity for rest and shortage of technicians, respectively. The total ranking of stressors in PHE staff regardless of the main categories showed that the most common stressors in this profession include „care of patients with critical health conditions, high number of missions, blaming of yourself when arriving late, fear of making mistakes in duty and care of patients who do not cooperate” (Figure 5(Fig. 5)).

Consistency index for each of the mean number matrixes (m) and the geometric mean of the upper and lower limits (g) for occupational stressors among nurses and PHE is shown in Table 5(Tab. 5). According to this table, the consistency rate for two matrixes, m and g was obtained less than 0.1. As a result, the fuzzy AHP questionnaire related to nurses and emergency personnel is valid and the final pairwise matrix comparison is compatible.

## Discussion

The general purpose of this study was to evaluate and rank occupational stressors in nurses and PHE staff using fuzzy hierarchical method. In this study, five categories of factors affecting the incidence of occupational stress in nurses and PHE staff (managerial, patient care, personal, interpersonal, interpersonal and environmental communication factors) were studied.

The results showed that the highest and lowest weight of stressors in nurses were related to managerial and environmental factors, respectively. Torshizi and Ahmadi (2011[52]) also showed that the most important occupational stressors among Iranian clinical nurses (Tehran) are managerial factors. Among managerial factors, „insufficient pay”, is the first priority in nurses which is consistent with the results of a previous study performed by Shojaei et al. (2013[48]).

Among environmental and interpersonal factors the highest weight was related to „lack of opportunity to rest“ and „being criticized by supervisors in the presence of others“, respectively. 

„Cardiopulmonary arrest of patient“ was the most important factor among the patient care stress factors. This finding is not in line with Lambert's study (Lambert et al., 2004[29]) that showed „Watching a patient suffer” was the most important item in this category. As a result, the most important factor in terms of „interpersonal stressors“ in nurses was recognized as „incompatibility between work schedule and life conditions”. 

Prioritizing occupational stressors in nurses regardless of the main categories of stressors, showed that „incompatibility between work schedule and life conditions”, „being criticized by supervisors in the presence of others“, „lack of interest to work in the current ward“, „inability to making decisions in emergency situations“ and „cardiopulmonary arrest of patients“ had the most important role in developing occupational stress among nurses, respectively. Various prioritizing studies have been carried out regarding the severity and importance of stressors. Data obtained from the study of Wang and Kong (2011[56]) in a surgical unit in Hong Kong, showed that „workload“, „lack of support“, „inadequate preparation” and „conflict with other nurses” are the main stressor resources among nurses. In the study of Tyson et al. (2002[54]) „inadequate organizational support“, „misunderstand the real needs of the ward by managers”, „conflicts with managers“, „shortage of personnel“, „increased workload due to staff shortage“, „job insecurity“ and „conflict between work and home activities“ were recognized as the most important occupational stress resources among nurses. Similarly, in the review study conducted by Sveinsdóttir et al. (2006[49]) on 522 people of the Nurses Association of Iceland, „heavy workload“, „inadequate consultation and communication“, „lack of performance feedback“, „inadequate source for work“ and „interference work with home chores“, were the main sources of stress in nurses.

In the PHE staff, „patient care“ and „managerial“ factors had the highest and lowest weight, respectively. Unlike nurses, in PHE staff, „managerial factors“ had the lowest priority among occupational stressors which is probably due to differences in the nature and working conditions of these two occupational groups. Patient care was the most important stressor in PHE staff. This is inconsistent with the findings of Motie et al. (2010[37]) and Hawley (1992[18]) that showed managerial agents as the most important stressor in PHE staff (Hawley, 1992[18]; Motie et al., 2010[37]). It seems that different study methods are the main cause of this difference. Meanwhile many of the sub-dimensions in the category of „patient care stressors“ are rooted in managerial factors. PHE technicians have the primary role in emergencies to get the patient to the physician. Therefore, they are encountered with a variety of stressors associated with patient care until they get to the physician. Among the management factors affecting the incidence of occupational stress in PHE staff, „shortage of technicians for mission“ was of the highest score. Among the stressors related to patient care, „care of patients with critical health conditions“ was of the highest score. Among individual factors of stress, „fear of making a mistake” earned the highest score. Among environment factors, „lack of opportunity to rest“ and finally among interpersonal stressors, „intervention of patient's companions in emergency care” were the most stressful factors.

In PHE technicians, regardless of main stress categories, „care of patients with critical health conditions”, „high number of missions”, „fear of making mistakes in duty”, „care of patients who do not cooperate” were the main stress factors, respectively. Some of these factors (e.g. shortage of personnel and resources) have been reported in other studies (Hawley, 1992[18]; Nirel et al., 2008[40]; Motie et al., 2010[37]) and some others are reported in this study.

The difference in the results of the studies performed regarding the stress factors in nurses and PHE staff in Iran and other countries indicates that stressors vary depending on the management system in each country and region, cultural condition, number of patients, facilities and physical structure of the hospital.

The final goal of identifying and ranking of occupational stressors is determining the optimal strategies to reduce occupational stress in nurses and PHE staff. From stress management perspective, stressors in nurses can be divided into two general categories including individual factors and factors related to management and organization. A combination of effective management practices and individual coping strategies is required for stress management of nurses and PHE staff. 

Management practices and organizational interventions for stress management are those efforts and rules set to manage stress among nurses and PHE staff. According to the results of the study, most occupational stress among nurses and PHE staff, directly or indirectly, was related to managerial and organizational stressors. Therefore, it seems that taking measures at the organization level is a most effective strategy to reduce the stress of nurses and PHE staff. However, success in implementing these strategies largely depends on the participation of employees. According to the results of the study, the following organizational strategies are suggested to protect nurses and PHE staff against the harmful consequence of occupational stress:

- Designing the payroll system and matching it with the workload of nurses and PHE staff;
- Modifying work practices and design of work-rest system through effective interpersonal communication;
- Developing clear professional roles and improved organizational climate through communication, social support, shared vision, and feedback;
- Review the rotation of work shifts;
- Redesigning the work environment to reduce environmental stressors including ergonomic interventions in sit-stand workstations and office workstations (McHugh and Schaller, 1997[35]; Choobineh et al., 2012[10]), providing adequate place for rest, improving ventilation system, provide safe environment, etc.);
- Providing opportunities for social support through enhancing peer to peer or supervisory support;
- Providing the necessary resources and facilities and meeting staff needs;
- Providing opportunities to staff participation in decisions and reducing hierarchy;
- Implementing programs and strategies to reduce violence against nurses and PHE staff.

Despite the development and implementation of organizational interventions, the occupational exposure of nurses and PHE staff with some of the stressors is inevitable. Therefore, individual coping strategies and interventions are required to reduce occupational stress among nurses and PHE staff. Coping strategies means the individual skills and techniques applied by nurses or PHE staff to manage stressful situation including meditation techniques, self-controlling, problem solving techniques, training about coping with occupational stress, support and advice from a psychologist, passive attendance by psychologists.

## Conclusion

This study showed that managerial and patient care factors are the most important causes of occupational stress among nurses and PHE staff, respectively. With respect to the important role of nurses and PHE staff in health care management system, effective control measures are required to reduce and manage stress factors, in order to improve their performance and efficiency in saving lives. Therefore, it is possible to improve care quality in these two professions by organizing work environment properly and by enacting supportive laws associated with these two professions.

## Limitations

The use of experts (faculty) and staff with work experience above 5 years was one of the study limitations. Therefore, the results may not be generalizable to the paramedic, nurse-aid and other age categories. Moreover, mental mood and fatigue of PHE staff and nurses may have an impact on how to answer the questionnaire and it was out of the researcher's full control.

## Acknowledgements

This study was extracted from the approved project with the code of 94-01-42-9771 and was financially supported by Shiraz University of Medical Sciences, Shiraz, Iran. The authors wish to thank the Research Consultation Center (RCC) at Shiraz University of Medical Sciences for their invaluable assistance in editing this manuscript.

## Disclosure of conflicting interests

The authors declare that there is no conflict of interest to disclose.

## Figures and Tables

**Table 1:**
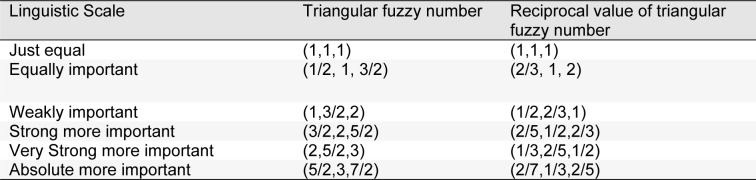
Linguistic Scale and corresponding fuzzy numbers

**Table 2:**
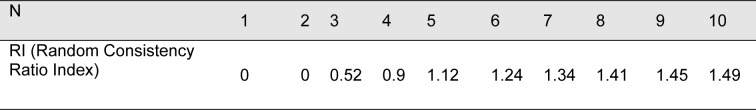
Inconsistency random matrix Index (Brunelli, 2014)

**Table 3:**
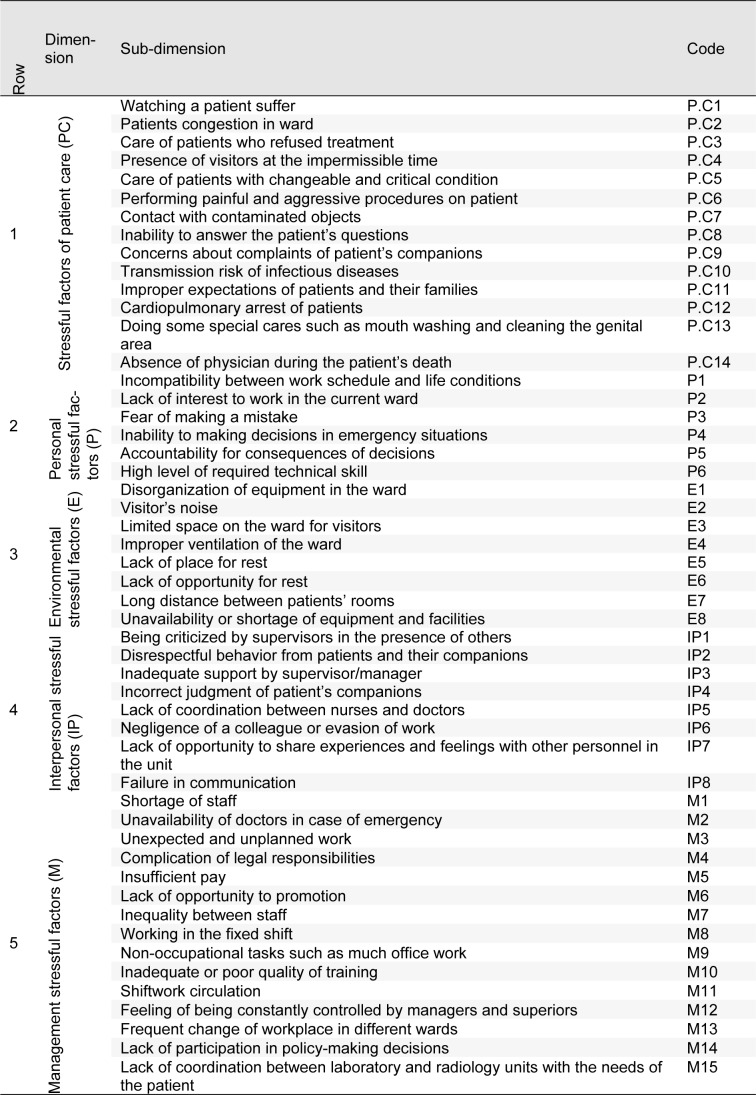
Identified dimensions and sub-dimensions of occupational stressors among nurses

**Table 4:**
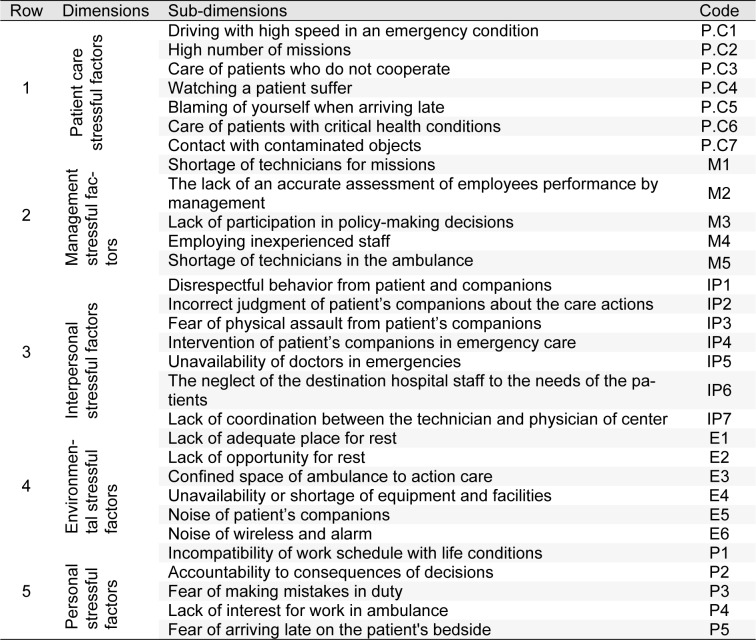
Identified dimensions and sub-dimensions of occupational stressors in pre-hospital emergency (PHE) staff

**Table 5:**
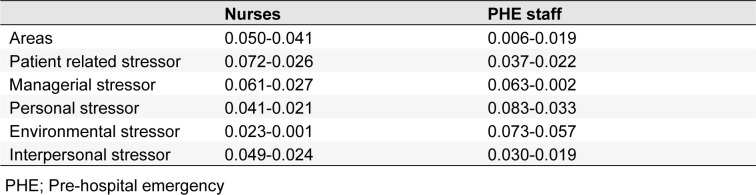
The values of consistency Ratio (CRm) for AHP paired comparisons matrix
